# Effects of Osteopathic Manipulative Treatment in a 3‐Year‐Old With Pitt‐Hopkins Syndrome

**DOI:** 10.1155/crpe/1433787

**Published:** 2026-06-17

**Authors:** Vaibhav Duggal, Rachel Radigan, Alexa Finkelstein, Sheldon Yao

**Affiliations:** ^1^ Department of Osteopathic Manipulative Medicine (OMM), New York Institute of Technology College of Osteopathic Medicine, Old Westbury, New York, USA, nyit.edu

## Abstract

Pitt‐Hopkins Syndrome (PTHS) is a rare neurodevelopmental disorder caused by a mutation in the transcription factor 4 gene resulting in defective regulation of the enteric nervous system. With no cure, current management emphasizes the treatment of the associated comorbidities to improve day‐to‐day quality of life. Osteopathic manipulative treatment (OMT) has been implemented in the treatment of various gastrointestinal pathologies; however, there is limited literature on its use in the management of comorbidities associated with PTHS. Here, we present a case of a three‐year‐old female with PTHS that greatly benefited from the use of five OMT sessions to resolve her complaints of severe constipation, abdominal pain, discomfort, and muscle spasms. As reported by the mother, the patient became less restless, had minimal abdominal pain, and was having multiple daily bowel movements with a reduction of her previous biweekly enema regimen. All while bringing attention to PTHS, this case demonstrates the efficacy of utilizing OMT to manage its gastrointestinal comorbidities in hopes of incorporating it into the standard of care.

## 1. Introduction

Pitt‐Hopkins Syndrome (PTHS) is a rare neurodevelopmental disorder first described in 1978, with an estimated incidence of 1:34,000 to 1:41,000 [1]. It results from pathogenic mutations in the transcription factor 4 (TCF4) gene located on chromosome 18 (18q21.2) [2]. TCF4 is a basic helix–loop–helix E‐protein essential for early brain development and neuronal differentiation. Animal studies demonstrate that heterozygous deletion of the DNA‐binding domain of TCF4 leads to gastrointestinal dysmotility and impaired enteric nervous system (ENS) function, highlighting its role in gut–brain regulation [3].

Clinically, PTHS is characterized by syndromic facies, psychomotor delay, and intellectual disability. Common physical findings include upslanting palpebral fissures, bitemporal narrowing, a wide nasal bridge, palmar creases, and microcephaly. Patients frequently present with multiple comorbidities such as chronic constipation (70%), early onset myopia (54%), strabismus (49%), apneic spells (48%), and epilepsy (38%), with many also meeting the criteria for autism spectrum disorder [4]. Neuroimaging is often nonspecific and may demonstrate cerebral atrophy, ventriculomegaly, and corpus callosum dysplasia [4].

There is currently no cure for PTHS, and management focuses on addressing comorbidities and neuropsychological difficulties while emerging therapies target the underlying TCF4 mutation. Gastrointestinal complications are among the most burdensome and can lead to severe outcomes, including aerophagia [1]. Given the prevalence of gastrointestinal dysmotility, osteopathic manipulative treatment (OMT) has been considered as a supportive therapeutic modality. OMT is a hands‐on, holistic approach aimed at restoring physiologic balance and enhancing the body’s inherent self‐regulatory mechanisms [5]. Prior literature reports improvement in chronic constipation among pediatric PTHS patients following structured OMT interventions, though published data remain limited [2]. Broader pediatric literature supports the safety and potential efficacy of OMT for a range of conditions, with systematic and scoping reviews demonstrating benefit across multiple functional and neuromusculoskeletal presentations [6–8].

This case demonstrates the potential role of OMT in improving severe constipation, abdominal discomfort, and pain in a pediatric patient with PTHS, supporting the need for broader investigation into its integration within multidisciplinary care strategies.

## 2. Case Presentation

### 2.1. Patient Information

A three‐year‐old female with a past medical history of PTHS due to de novo genetic mutation presented with severe constipation, terrible bouts of abdominal pain, built‐up gas, muscle spasms, and gastrointestinal issues. The mother had a complicated pregnancy with the patient due to small gestational age, small head circumference, minimal movement, and low tone. The patient was born via C‐section at 37.5 weeks and remained in the NICU for 1 week after birth.

The patient is nonverbal and nonambulatory, so her history was provided by her mother who had read about the use of OMT for constipation. She stated that the patient has severe constipation, restlessness, and pain about 50% of the time. The patient was currently on a regimen of MiraLAX daily and an enema given every 2 weeks in order to aid in the relief of her constipation without significant improvement. After looking into OMT for relief of gastrointestinal issues, the patient’s mother decided to bring her daughter for treatment.

### 2.2. Clinical Findings

On initial presentation, the patient’s vitals were stable and insignificant except for a BMI of 15. Her temperature was 97.9F, pulse oximetry was 98%, and respiratory rate was 20/min. She appeared nervous and required distractions to remain still throughout the visit. Her abdominal exam was significant for mild distention but was soft with bowel sounds present and no rebound tenderness, rigidity, or guarding. Her neurological exam was significant for inability to form words, needing full assistance for transfer from stroller to table as well as being unable to stand or walk by herself. However, she was able to maintain posture while sitting and can move all four extremities spontaneously.

Osteopathic structural exam was significant for right occipitomastoid compression, celiac ganglion restriction, ascending colon density, left lower rib exhalation dysfunctions, bilateral psoas spasms, a left posterior innominate, and an anterior sacral base. The occipitoatlantal (OA) junction was flexed, side‐bent left, and rotated right; C3 was flexed, side bent left, and rotated left; and C5 was extended side bent and rotated right. Further restrictions throughout the thoracic vertebrae were also found.

### 2.3. Timeline

### 2.4. Diagnostic Assessment

Outcomes were based on both subjective observations by the patients’ caregivers and objective observations noted by the physician. Improvements were primarily reported through caregiver observations. Retrospectively, the pediatric face, legs, activity, cry, consolability (FLACC) scale was applied to objectively assess outcomes in our nonverbal patient. The FLACC scale has been shown to be a reliable and valid tool for evaluating pain in children who are unable to self‐report, and its ease of use makes it particularly valuable for routine clinical assessment of pediatric discomfort [9].

### 2.5. Therapeutic Intervention

Consent was obtained from the patient’s mother, and then the patient began weekly visits for 2 weeks and biweekly for three additional treatments. Therapeutic intervention consisted of patient‐targeted OMT. Each OMT session consisted of various techniques including OA decompression, thoracic inlet release, abdominal diaphragm doming, visceral techniques targeting the gastrointestinal system and the abdomen, rib raising, paraspinal muscle inhibition, and articulatory techniques for the sacrum and pelvis. In order to address the specific gastrointestinal complaints secondary to PTHS counterstrain to the ileocecal valve and gastroduodenal junction, mesenteric ganglion inhibition and colonic stimulation were performed. The latter two were taught to the patient’s mother as well, allowing her to perform them daily as needed. Subsequent visits followed the same format of utilized techniques.

### 2.6. Follow‐Up and Outcomes

The patients’ behaviors and outcomes are reported from the caregiver’s perspective. The patient’s mother reported that her daughter was much more relaxed after each session, being able to nap after lunch which was uncommon previously.

“She immediately fell asleep in the car back to school today and apparently needed a nap after lunch which virtually never happens. I wonder if the treatment contributed to that? (Relaxed her etc.?)”

She also stated that her daughter seemed happier, less uncomfortable, and was having multiple daily bowel movements. Due to the increased naturally occurring bowel movements, the patient required less rectal stimulations. She did, however, report her daughter’s occasional struggle with being gassy where she appeared uncomfortable.

“[She] is doing OK. She has good days and bad days and while that’s usually dependent on her GI issues I’m not so sure lately what the issue is. She’s been very tired lately and sleeping a lot more so that’s a change I’ve noticed. We’re definitely doing less rectal stimulations (more naturally occurring BMs) so that’s also something I’ve noticed since starting the therapies.”

Eight weeks after her daughter’s last OMT visit, the patient’s mother stated that her daughter’s gastrointestinal symptoms were still much improved and well controlled, with her being in an overall better mood.

“[She] is doing OK she has good and bad days more good lately I’d say pooping seems better.”

**FIGURE 1:**
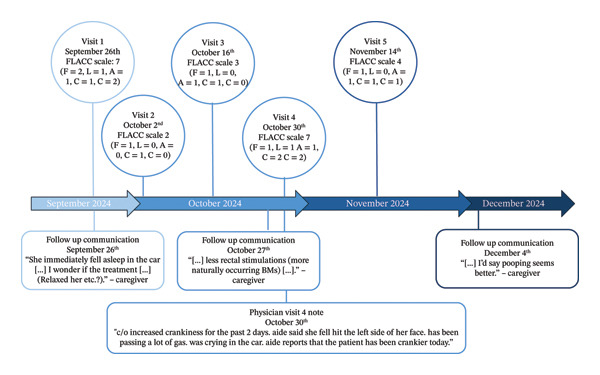
Timeline of patient care and outcomes: patients’ visit dates with retrospective physician scored pediatric face, legs, activity, cry, consolability (FLACC) scale for nonverbal and abridged versions of caregiver outcome quotes.

The attending physician observed progressive improvement in somatic dysfunctions and temperament, with the patient remaining calmer each and able to stay on the table without electronic distractions. Retrospective assessment using the FLACC scale confirmed these findings, with scores decreasing from 7 at visit one to 2 at visit two and remaining below the patients’ starting score, with a score of 4 at visit five (Figure 1). Throughout treatment, OMT was well tolerated with no adverse effects.

## 3. Discussion

PTHS is a complex neurodevelopmental frequently associated with significant gastrointestinal dysfunction. The genetic mutation of the TCF4 is believed to impair regulation of the ENS, resulting in reduced gut propulsive motility and subsequent symptoms such as constipation, abdominal pain, bloating, and discomfort [2]. These manifestations highlight the importance of the brain–gut axis in PTHS pathophysiology.

The brain–gut connection is mediated through both autonomic and enteric pathways. The autonomic nervous system, particularly the vagus nerve, provides bidirectional communication between the central nervous system and gastrointestinal tract. The vagus nerve innervates mucosal and muscular layers of the gut, transmitting sensory input related to luminal pressure, distention, and inflammation while modulating efferent responses influencing motility, secretion, and immune function [10]. A second critical component is the ENS, consisting of over one hundred million neurons, which communicates with peripheral ganglia, the vagus nerve, and central neural structures such as the nucleus tractus solitarius. Disruption of this complex network in PTHS likely underpins the severe and persistent gastrointestinal symptoms observed in affected individuals [11].

OMT has been demonstrated to restore autonomic balance and enhance visceral mobility, supporting its application in managing gastrointestinal disorders [5]. In a prior case study involving a pediatric patient with PTHS, six weekly OMT sessions resulted in increased bowel movements and reduced need for frequent enemas [2]. In broader pediatric populations, OMT has shown benefit in improving constipation, abdominal pain, and overall quality of life in children with irritable bowel syndrome and other functional GI disorders [12–14]. Systematic and scoping reviews further support the feasibility and therapeutic value of OMT in pediatric populations and complex neurological conditions, reinforcing its role as an adjunctive modality in multidisciplinary care [6–8, 15]. Recent case‐based literature also demonstrates the application of osteopathic treatment in neurologically complex patients, including individuals with Arnold–Chiari malformation, syringomyelia, and fetal alcohol spectrum disorder, highlighting its expanding role in addressing neurocognitive and functional symptoms [16, 17].

In the present case, treatment was implemented through two primary approaches: first, autonomic rebalancing targeting parasympathetic and sympathetic regulation using OA decompression to influence vagal tone, sacral and innominate techniques to address sacral nerve roots, and rib raising and paraspinal inhibition to reduce sympathetic hyperactivity; second, efforts focused on improving fascial mobility and visceral motion through mesenteric release, counterstrain to the ileocecal valve and gastroduodenal junction, and techniques addressing thoracic inlet and abdominal restriction to support lymphatic drainage and mechanical function [18]. Additionally, caregiver education in colonic stimulation and mesenteric release empowered ongoing symptom management at home.

This case study has several notable limitations. As a single‐patient report, it lacks a control group and cannot establish causality or generalizability. With a single patient, there was variability and fluctuations between visits, but overall subjective and objective improvements were still noted. Objective outcome measures were limited, with only retrospective FLACC scale assessments performed, resulting in reliance primarily on caregiver‐reported symptom improvement rather than standardized gastrointestinal scoring systems or validated quality‐of‐life metrics. Additionally, follow‐up duration was relatively short, precluding assessment of long‐term efficacy and sustainability of symptom improvement. Potential placebo effects and concurrent interventions could not be fully controlled.

PTHS is a rare, complex syndrome where treatment is often multimodal. With no current cure, the best course is management of comorbidities and symptoms, often gastrointestinal in nature. Our case demonstrates measurable clinical improvement in gastrointestinal symptoms after OMT, suggesting that OMT may serve as a beneficial adjunctive therapy within a multidisciplinary care framework. Future investigations should include controlled studies with larger patient populations, incorporation of validated objective outcome measures (e.g., stool frequency scales, pain scores, and GI motility assessments), and longer‐term follow‐up to better evaluate the role of OMT in PTHS management. Expanding research in this area may contribute to establishing standardized, evidence‐based guidelines for managing gastrointestinal comorbidities in this rare population.

## 4. Conclusion

PHS is a rare disorder with limited evidence‐based management strategies. In this case, OMT was associated with improvements in severe constipation, abdominal discomfort, and pain, suggesting its potential as an adjunctive therapy within multidisciplinary care. These findings contribute to the emerging, yet sparse, body of literature addressing integrative approaches for symptom management in this population. However, as a single‐case report with subjective outcomes and limited follow‐up, these findings cannot be generalized. Future studies should employ larger, controlled designs with standardized, objective measures to clarify the efficacy and clinical role of OMT in managing gastrointestinal symptoms in PHS.

## Funding

The authors have no financial interest to declare in relation to the content of this article.

## Consent

The patient’s mother provided written informed consent prior to participation.

## Conflicts of Interest

The authors declare no conflicts of interest.

## Data Availability

The data that support the findings of this study are available on request from the corresponding author. The data are not publicly available due to privacy or ethical restrictions.

## References

[1] Koppen I. J. N. , Menke L. A. , Westra W. M. et al., Fatal Gastrointestinal Complications in Pitt-Hopkins Syndrome, American Journal of Medical Genetics, Part A. (2023) 191, no. 3, 855–858, 10.1002/ajmg.a.63079. 36511359

[2] Aquino A. , Perini M. , Cosmai S. et al., Osteopathic Manipulative Treatment Limits Chronic Constipation in a Child With Pitt-Hopkins Syndrome, Case Reports in Pediatrics. (2017) 2017, no. 1, 10.1155/2017/5437830. PMC5306969 28251008

[3] Grubišić V. , Kennedy A. J. , Sweatt J. D. , and Parpura V. , Pitt-Hopkins Mouse Model Has Altered Particular Gastrointestinal Transits in Vivo, Autism Research. (2015) 8, no. 5, 629–633, 10.1002/aur.1467. 25728630 PMC5724775

[4] Tan A. , Goodspeed K. , and Edgar V. B. , Pitt-Hopkins Syndrome: a Unique Case Study, Journal of the International Neuropsychological Society. (2018) 24, no. 9, 995–1002, 10.1017/S1355617718000668. 30375316

[5] Roberts A. , Harris K. , Outen B. et al., Osteopathic Manipulative Medicine: A Brief Review of the Hands-On Treatment Approaches and Their Therapeutic Uses, Medicine. (2022) 9, no. 5, 10.3390/medicines9050033. PMC9143587 35622072

[6] Franke H. , Franke J. D. , and Fryer G. , Effectiveness of Osteopathic Manipulative Treatment for Pediatric Conditions: A Systematic Review, Journal of Bodywork and Movement Therapies. (2022) 31, 113–133, 10.1016/j.jbmt.2022.03.013. 35710210

[7] DeMarsh S. , Huntzinger A. , Gehred A. , Stanek J. R. , Kemper K. J. , and Belsky J. A. , Pediatric Osteopathic Manipulative Medicine: A Scoping Review, Pediatrics. (2021) 147, no. 2, 10.1542/peds.2020-016162. 33500321

[8] Parnell Prevost C. , Gleberzon B. , Carleo B. , Anderson K. , Cark M. , and Pohlman K. A. , Manual Therapy for the Pediatric Population: A Systematic Review, BMC Complementary and Alternative Medicine. (2019) 19, no. 1, 10.1186/s12906-019-2447-2. PMC6417069 30866915

[9] Crellin D. J. , Harrison D. , Santamaria N. , and Babl F. E. , Systematic Review of the Face, Legs, Activity, Cry and Consolability Scale for Assessing Pain in Infants and Children: Is it Reliable, Valid, and Feasible for Use?, Pain. (2015) 156, no. 11, 2132–2151, 10.1097/j.pain.0000000000000305. 26207651

[10] Gwak M. G. and Chang S. Y. , Gut-Brain Connection: Microbiome, Gut Barrier, and Environmental Sensors, Immune Network. (2021) 21, no. 3, 10.4110/in.2021.21.e20. PMC8263213 34277110

[11] Rao M. and Gershon M. D. , The Bowel and Beyond: The Enteric Nervous System in Neurological Disorders, Nature Reviews Gastroenterology & Hepatology. (2016) 13, no. 9, 517–528, 10.1038/nrgastro.2016.107. 27435372 PMC5005185

[12] Attali T. V. , Bouchoucha M. , and Benamouzig R. , Treatment of Refractory Irritable Bowel Syndrome With Visceral Osteopathy: Short-Term and Long-Term Results of a Randomized Trial, Journal of Digestive Diseases. (2013) 14, no. 12, 654–661, 10.1111/1751-2980.12098. 23981319

[13] Stiedl M. , Müller A. , Salomon J. , Schwerla F. , and Resch K. , Osteopathy as a Promising Short-Term Strategy for Irritable Bowel Syndrome: Randomized Controlled Trial, Focus on Alternative and Complementary Therapies. (2010) 8, no. 4, 10.1111/j.2042-7166.2003.tb04066.x.

[14] Hundscheid H. W. , Pepels M. J. , Engels L. G. , and Loffeld R. J. , Treatment of Irritable Bowel Syndrome With Osteopathy: Results of a Randomized Controlled Pilot Study, Journal of Gastroenterology and Hepatology. (2007) 22, no. 9, 1394–1398, 10.1111/j.1440-1746.2006.04741.x. 17716344

[15] Cerritelli F. , Ruffini N. , Lacorte E. , and Vanacore N. , Osteopathic Manipulative Treatment in Neurological Diseases: Systematic Review, Journal of the Neurological Sciences. (2016) 369, 333–341, 10.1016/j.jns.2016.08.062. 27653920

[16] Cases-Solé R. , Varillas-Delgado D. , Trinidad-Cascudo F. , Pino-Tamayo M. C. , and García-Algar Ó. , Osteopathic Treatment of a Person With Arnold–Chiari Malformation and Syringomyelia: A Case Report, International Journal of Osteopathic Medicine. (2024) 54, 10.1016/j.ijosm.2024.100730.

[17] Cases-Solé R. , Varillas-Delgado D. , Astals-Vizcaino M. , and García-Algar Ó. , Efficacy and Effectiveness of Osteopathic Intervention for Neurocognitive and Behavioral Symptoms Associated With Fetal Alcohol Spectrum Disorder, Frontiers in Behavioral Neuroscience. (2022) 16, 10.3389/fnbeh.2022.860223. PMC8965441 35368309

[18] Nicholas A. S. and Nicholas E. A. , Atlas of Osteopathic Techniques, 2023, 4th edition, Lippincott Williams & Wilkins.

